# A self‐checking treatment couch coordinate calculation system in radiotherapy

**DOI:** 10.1002/acm2.12771

**Published:** 2019-11-18

**Authors:** Pingfang Tsai, Chihray Liu, Darren L. Kahler, Jonathan G. Li, Bo Lu, Guanghua Yan

**Affiliations:** ^1^ Department of Radiation Oncology University of Florida College of Medicine Gainesville FL USA

**Keywords:** patient setup, couch coordinate, workflow automation, error prevention

## Abstract

**Purpose:**

Traditionally, the treatment couch coordinates (TCCs) for patients undergoing radiotherapy can only be determined at the time of treatment, placing pressure on the treating therapists and leaving several pathways for errors such as wrong‐site treatment or wrong treatment table shift from a reference point. The purpose of this work is to propose an accurate, robust, and streamlined system that calculates TCC in advance.

**Methods:**

The proposed system combines the advantages of two different calculation methods that use an indexed immobilization device. The first method uses an array of reference ball bearings (BBs) embedded in the CT scanner’s couch‐top. To obtain the patient‐specific TCC, the spatial offset of the treatment planning isocenter from the reference BB is used. The second method performs a calculation using the one‐to‐one mapping relationship between the CT scanner’s DICOM (Digital Imaging and Communications in Medicine) coordinate system and the TCC system. Both methods use a reference point in the CT coordinate system to correlate a point in the TCC system to perform the coordinate transfer between the two systems. Both methods were used to calculate the TCC and the results were checked against each other, creating an integrated workflow via automated self‐checking. The accuracy of the calculation system was retrospectively evaluated with 275 patients, where the actual treatment position determined with cone‐beam CT was used as a reference.

**Results:**

An efficient workflow transparent to the therapists at both CT simulation and treatment was created. It works with any indexed immobilization device and can be universally applied to all treatment sites. The two methods had comparable accuracy, with 95% of the calculations within 3 mm. The inter‐fraction variation was within ± 1.0 cm for 95% of the coordinates across all the treatment sites.

**Conclusions:**

A robust, accurate, and streamlined system was implemented to calculate TCCs in advance. It eases the pressure on the treating therapists, reduces patient setup time, and enhances the patient safety by preventing setup errors.

## 1 INTRODUCTION

Radiation therapists are usually under pressure when starting a new patient’s treatment. It is critical to them to correctly position a patient on a treatment couch and move it to the treatment location. Traditionally, therapists rely on skin tattoos (reference marks) made on a patient during CT simulation and shift instructions provided by the computerized‐treatment planning system (TPS) to initiate the task. A patient is first positioned by aligning the tattoos to room lasers and is then moved with the treatment couch according to the shift instructions. It has been shown that there are several pathways that can lead to wrong‐site treatment errors.^1^, ^2^, ^3^ For example, based on the analysis of the incidences reported to the Radiation Oncology Incident Learning System (RO‐ILS), Ezzell et al.^4^ found that 18% of the high priority events were attributable to either wrong shift instructions or a wrong shift performed during the treatment. Other common pathways include (a) the patient was marked incorrectly at CT simulation, (b) the patient marks were incorrectly identified in the TPS, (c) the reference image set for image‐guided radiation therapy (IGRT) was created with the wrong isocenter or the wrong dataset. Given the number of common pathways leading to wrong‐site treatment errors, there is an urgent need for automating the patient setup process to smooth out the workflow, mitigate the pressure on the therapists, and strengthen the first line of defense against such errors. A critical piece of the automation, referred to in this work, is the determination of treatment couch coordinates (TCCs) before the patient is even put onto the treatment couch. It is highly desirable to have a simple system to calculate the couch coordinates prior to the commencement of the actual treatment.

The increasing use of indexed immobilization devices presents an opportunity for such automation. Indexed immobilization devices not only enable the patients to reproduce and maintain their positions from CT simulation throughout the course of the treatment, they also provide a mechanism to relate patient position to treatment table position through indexing. Saenz et al.^5^ reported a method for patient‐specific couch coordinate prediction using indexed immobilization devices. Their method relied on a radiographically apparent landmark on the immobilization device. The baseline couch coordinates (the couch coordinates when the landmark was positioned at treatment room isocenter) were first determined. Then the displacement of the planned treatment isocenter relative to the landmark was used to adjust the baseline table coordinates to obtain the TCCs for the patient. Overall, 86% of their predictions were correct to within 2 cm and the mean error was under 0.1 cm, but the standard deviation was relatively large (1.47 cm). Additionally, their method was dictated by the immobilization device. For devices without a radiographically apparent landmark, the method failed to predict couch coordinates. Sueyoshi et al.^6^ reported a slightly different approach. Instead of using landmarks on the immobilization device, they used a set of fixed couch‐top positions (e.g., indexing notches within the couch‐top) as the reference. Their method can be applied with any indexed immobilization device, as it is not immobilization device‐specific. The authors pointed out that their method, combined with a surface‐guided imaging system AlignRT® (VisionRT, London, UK), was effective in eliminating wrong‐site treatment mistakes. However, no quantitative analysis of the method’s accuracy was provided in their report. Additionally, their workflow is not efficient, as it consists of several steps requiring human involvement in decision‐making. Several other groups^7^, ^8^, ^9^, ^10^ reported efforts to detect setup mistakes by tightening the site‐specific tolerances for the couch coordinates. These methods, commonly known as tolerance table approaches, do not calculate the table coordinates in advance. Therefore, the onus remains on the therapists to figure them out while the patient is on the treatment table.

In our opinion, an ideal method to automate the initial patient setup should have the following features: (a) it calculates couch coordinates in advance to ease the pressure on the therapists; (b) it is universally applicable to all treatment sites; (c) it is not immobilization device‐specific; in other words, it works with any indexed immobilization device; (d) it offers a smooth workflow by reducing or simplifying intermediate steps; (e) it has simple, yet rigorous, quality assurance (QA) measures to ensure its integrity. The aim of this paper was to present a patient setup automation solution to accomplish the above goals. We first presented two independent TCC calculation methods then introduced a highly automated, self‐checking system that seamlessly integrated the two methods.

## 2 METHODS

### 2.1 Reference BB‐based calculation method

The main idea of this method is to relate the treatment planning isocenter to the reference ball bearings (BBs) that are embedded in the CT couch‐top. The reference couch coordinates (RCCs), that is, the couch coordinates to position the point representing a primary reference BB at treatment room isocenter, are predetermined. The patient‐specific TCCs are calculated by adjusting the RCCs according to the displacement between the treatment planning isocenter and the primary reference BB. For this method to work, an indexed immobilization device is used to maintain the spatial relationship between the patient and the couch‐top. With this method, the TCCs are calculated at the treatment planning stage, significantly reducing the burden on the therapists at the time of treatment.

**Figure 1:**
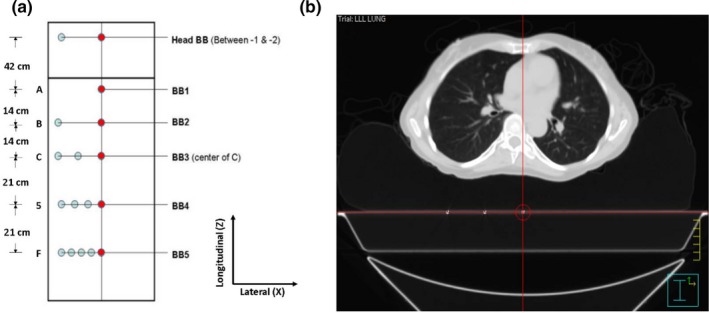
Ball bearings embedded couch design. (a) The arrangement of ball bearings (BBs) embedded in a CT couch‐top. The BBs on the midline are used as references for couch coordinate calculations; others are used to facilitate the reference marker identification. (b) An axial CT slice passes through a reference BB (BB_3_) selected as the laser coordinate system origin in the treatment planning system.

Figure 1(a) depicts the arrangement of the BBs embedded in the CT couch‐top. All the BBs are flush with the couch surface. There are 17 BBs in six rows. In each row, the rightmost BB (referred to as reference BB) is placed on the midline of the couch, and the others are arranged to the left of the midline when viewed from above the couch. There are two BBs in the most superior row, with the reference BB referred to as the “Head BB.” The number of BBs varies in the other rows, where the reference BB is referred to as “BB1,” “BB2,” “BB3,” “BB4,” or “BB5”, with the number indicating the number of BBs in each row. This pattern makes it easy to identify the reference BBs on a CT scan. For example, the reference BB shown in [Fig 1(b)] can be recognized as BB3 since there are three BBs in the row. Both the Head BB row and the BB2 row have two BBs. They can be distinguished by the fact that, generally, the Head BB only shows up in a head/brain scan, whereas BB2 only shows up in a lung/chest scan.

At our institution, we use an iBeam® EVO couch‐top (Elekta Inc., Stockholm, Sweden) with a Phillip Brilliance Big Bore CT simulator (Phillips Medical Systems, Madison, WI). The same couch‐top is used in each treatment room. The indexing holes on the couch‐top are labeled with a single digit or letter [Fig. 1(a)]. BB3, designated as the primary reference BB, is located midway between the pair of “C” indexing holes. The longitudinal distance between each adjacent reference BB is known, as shown in [Fig. 1(a)], from which the longitudinal offset (OS_BBi,z_) of the *i*th reference BB with respect to BB3 can be determined. The vertical offset (OS_BBi,y_) and lateral offset (OS_BBi,x_) are both zero since all the reference BBs are in the same coronal and saggital planes. The RCCs for BB3 (RCC_BB3_) are determined as the couch coordinates when the treatment couch is positioned such that the midpoint of the two “C” indexing holes is aligned with the treatment room isocenter.

**Figure 2:**
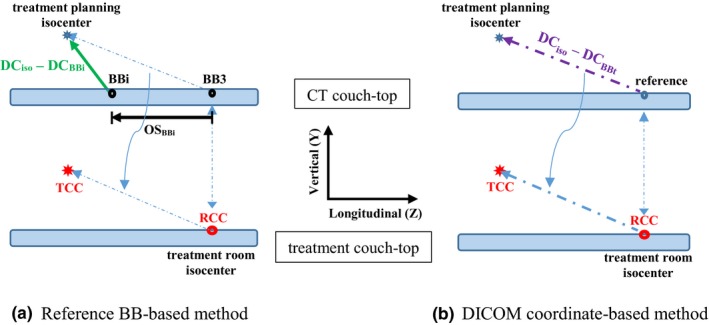
The self‐checking treatment couch coordinate (TCC) calculation system with two methods. (a) Illustration of the reference BB‐based TCC calculation method. The treatment planning isocenter is related to a reference BB (BBi), which in turn is related to the primary reference BB (BB3). The displacement vector from the treatment planning isocenter to BB3 is used to adapt the reference couch coordinates (RCCs) to obtain patient‐specific TCCs. (b) Illustration of the DICOM coordinate‐based TCC calculation method. The one‐to‐one mapping relationship from the CT DICOM coordinates to the TCCs is established with a reference point. The displacement vector from the treatment planning isocenter to the reference is used to adapt the RCCs to obtain TCCs.

The Pinnacle TPS (Philips Radiation Oncology Systems, Fitchburg, WI) is used at our institution. During treatment planning, the reference BB closest to the treatment area is selected as the laser coordinate system origin in the CT dataset. The arrangement of the BBs on the couch‐top guarantees that at least one reference BB is included in each patient’s CT scan. A point of interest (POI) is created at the location of the selected reference BB. Then the DICOM (Digital Imaging and Communications in Medicine) coordinates of this POI (DC_BBi_) and the planned treatment isocenter (DC_iso_) are transferred from the TPS to an in‐house program to calculate the TCCs. The TCCs in the lateral (TCC_x_), vertical (TCC_y_), and longitudinal (TCC_z_) directions for the patient are calculated as.

$${\text{TCC}}_{x}={\text{RCC}}_{\text{BB3},x}+{\text{OS}}_{\text{BBi},x}+{\text{DC}}_{\text{iso},x}-{\text{DC}}_{\text{BBi},x}$$

$${\text{TCC}}_{y}={\text{RCC}}_{\text{BB}3,y}+{\text{OS}}_{\text{BBi},y}+{\text{DC}}_{\text{iso},y}-{\text{DC}}_{\text{BBi},y}$$

$${\text{TCC}}_{z}={\text{RCC}}_{\text{BB}3,z}+{\text{OS}}_{\text{BBi},z}+{\text{DC}}_{\text{iso},z}-{\text{DC}}_{\text{BBi},z}$$

respectively. OS_BBi,x_ and OS_BBi,y_ can be omitted in the equations since they are both zero. It is assumed that the indexing bars of the indexed immobilization device are placed at the default locations (holes). If nondefault indexing locations need to be used for clinical reasons (e.g., to avoid collision), the corresponding longitudinal offset needs to be accounted for in the equation for TCC_z_. Figure 2(a) illustrates the relationship between the treatment planning isocenter and the reference BBs. These TCCs are manually entered into the MOSAIQ® Record‐and‐Verify (R&V) system (Elekta Inc., Stockholm, Sweden) for each treatment beam, which are checked later during initial chart check. If multiple isocenters are used in the treatment plan, the above process is repeated for each treatment isocenter.

For the in‐house program to apply the correct longitudinal offset (OS_BBi,z_), the user needs to specify which reference BB has been selected in the TPS. Incorrect specification leads to a calculation error of 14 cm or more in the longitudinal direction [Fig. 1(a)]. To avoid such errors, the axial CT slice passing through the selected reference BB is recorded as a screenshot from the TPS, and a report detailing the user’s reference BB specification is generated by the in‐house program. These two documents are cross‐checked by a physicist during the initial chart check.

Although these types of errors can be detected by the physicist during chart checking, it is desirable to eliminate the source of error by automating the workflow. The automation can be achieved by using the CT scanner’s DICOM coordinate system, which will be discussed in the following sections. First, we will introduce another calculation method that is based on CT DICOM coordinates and requires no reference BBs (therefore, no alteration to the couch‐top). Then a self‐checking system combining the advantages of the reference BB‐based calculation method and the DICOM coordinate‐based calculation method will be described.

### 2.2 DICOM coordinate‐based calculation method

The motivation for this method stems from an observation that we made after we clinically implemented the reference BB‐based method. The observation was that, even though the initial couch position for each CT scan varies between patients, the DICOM coordinates of the reference BBs in all of the CT scans have only small variations (within 1 cm) along any axis. Considerable changes are only observed in the longitudinal direction when the CT couch‐top coordinate system is changed, which occurs when the reset button on the scanner’s control panel is pressed. This observation indicates that the DICOM coordinate system of the CT scanner is defined with respect to the CT couch‐top. In other words, unless changed by the operator, the DICOM coordinate system (origin and axes) remains stationary in relation to the couch‐top. Therefore, there is a one‐to‐one mapping relationship between the CT DICOM coordinate system and the treatment couch coordinate system. The TCCs to position any point in a CT scan at the treatment room isocenter can be determined if we can do so for a reference point (e.g., a landmark on the couch‐top). Since the reference is only needed when establishing the mapping relationship, there is no need to permanently embed BBs into the couch‐top. We temporarily taped a BB (referred to as BB_t_) at the midpoint between the two “C” index holes on the couch‐top and removed it after taking a CT scan of the couch‐top, from which the DICOM coordinates of BB_t_ were determined as DC_BBt_. The RCCs for BBt, denoted as RCC_BBt_, were determined by positioning the midpoint between the two “C” index holes on the treatment couch‐top at the treatment room isocenter. If we denote the DICOM coordinates of the treatment planning isocenter by DC_iso_, the patient‐specific TCCs can be calculated as,

$${\text{TCC}}_{x}={\text{RCC}}_{\text{BBt},x}+{\text{DC}}_{\text{iso},x}-{\text{DC}}_{\text{BBt},x}$$

$${\text{TCC}}_{y}={\text{RCC}}_{\text{BBt},y}+{\text{DC}}_{\text{iso},y}-{\text{DC}}_{\text{BBt},y}$$

$${\text{TCC}}_{z}={\text{RCC}}_{\text{BBt},z}+{\text{DC}}_{\text{iso},z}-{\text{DC}}_{\text{BBt},z}$$

RCC_BBt_ and DC_BBt_ are not patient‐specific. As illustrated in [Fig. 2(b)], these equations map the DICOM coordinates of any point in a CT scan directly to the treatment room coordinates. This method also relies on the use of indexed immobilization devices. Consistent indexing holes should be used between CT scan and treatment. Otherwise, the longitudinal offset due to the difference in indexing holes needs to be accounted for in the equation for TCC_z_.

### 2.3 A self‐checking couch coordinate calculation system

The advantage of the DICOM coordinate‐based method over the reference BB‐based method is that it directly uses the DICOM coordinates and does not use reference BBs embedded in the CT couch‐top, which further simplifies the workflow. However, the DICOM coordinate system can be changed unknowingly during daily operation as the reset button on the control panel is located right next to other frequently used buttons (e.g., buttons that move the couch in, out, up and down). This leaves a pathway for potential errors. It is desirable to combine the advantages of the two methods to streamline the workflow and eliminate the error pathway.

Here we introduce a self‐checking treatment couch coordinate calculation system that combines the two methods. The reference BB‐based method requires the user to specify which reference BB has been selected in the TPS. This can be a potential error pathway as the user can potentially specify the reference BB incorrectly. The CT scanner’s DICOM coordinate system, unless reset, remains stationary in relation to the CT couch‐top. Therefore, the reference BBs can be automatically distinguished based on their DICOM coordinates. This feature can be used to automate the specification of the reference BB. To this end, a lookup table containing all of the reference BBs’ longitudinal DICOM coordinates was stored in the in‐house software. For individual patients, the selected reference BB’s longitudinal DICOM coordinate, exported from the TPS, is compared to the values in the lookup table to automatically determine which reference BB has been selected. This simplifies the workflow and eliminates the error pathway for incorrect reference BB specification. Additionally, the TCCs are calculated with both the reference BB‐based method and the DICOM coordinate‐based method. The TCCs are only manually entered into the R&V system when the numbers calculated by the two methods agree with each other to within a specified tolerance.

**Figure 3:**
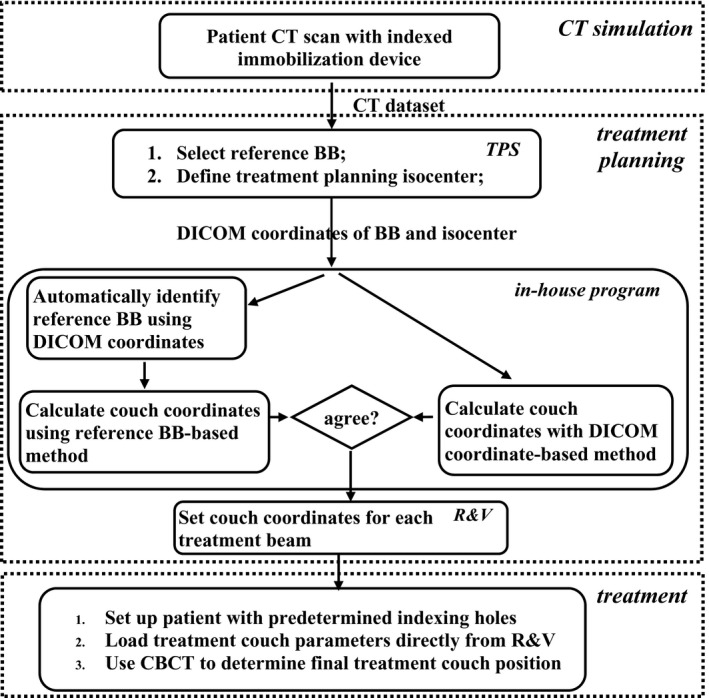
Workflow of the self‐checking couch coordinate calculation system.

The workflow for the combined method is illustrated in Fig. 3. At CT simulation, the therapists use predetermined indexing holes for the immobilization devices, which can be a full‐body Vac‐Loc^TM^ bag mold (CIVCO, Coralville, Iowa), a half body Vac‐Loc^TM^ mold, and breast boards or a Qfix AccuFix^TM^ device (Qfix, Avondale, PA) in our institution. During treatment planning, the dosimetrists pick a reference BB close to the treatment target and define a treatment planning isocenter. The DICOM coordinates of the reference BB and the planning isocenter are transferred from the TPS to the in‐house software. The in‐house software automatically detects which reference BB has been selected and applies the corresponding offset (OS_BBi, y_) to calculate the TCCs. The software also calculates the TCCs using the DICOM coordinate‐based method. It then performs a self‐checking process by comparing the TCCs calculated with the two methods. If the difference is within the specified tolerance, the results calculated with the reference BB‐based method are entered into the R&V system. During the treatment, the therapists use default indexing holes for the immobilization devices and load the TCCs directly from the R&V system to set up the patient. The final treatment position is determined using a cone‐beam tomography (CBCT). In our institution, daily CBCT is performed with the Elekta XVI linac‐integrated system (Elekta Inc., Stockholm, Sweden) for patient localization. The process to determine the TCCs is transparent to the therapists at both CT simulation and treatment.

### 2.4 Data collection and analysis

The variation of the CT scanner’s DICOM coordinate system from one CT scan to another was first evaluated without a patient on the treatment couch. The CT couch‐top, which can only move in the vertical and longitudinal directions, was scanned at various vertical positions covering its vertical range. At each vertical position, the couch‐top was moved to various longitudinal positions inside its moving range and the scan was repeated three times. The CT scans were sent to the TPS where BB3 was identified as a point and the variation of its DICOM coordinates was quantified.

The uncertainty of the DICOM coordinate system with a patient on the treatment couch was retrospectively analyzed with CT scans of patients. The longitudinal position of the couch‐top varies from patient to patient, depending on the anatomical location of the intended treatment area. The variation can cause different amounts of couch sag that adds to the uncertainty of the DICOM coordinates. To account for the effect, we included a wide variety of patients with diseases for different sites (e.g., brain, head and neck, lung, pelvis, etc.). For each patient, only the reference BB selected for TCC calculation was included in the analysis. In this way, the uncertainty analysis of the DICOM coordinates is relevant and meaningful to the purpose of this study.

The accuracy of the two calculation methods was evaluated by comparing the calculated TCCs with the ones determined using CBCT, which were retrospectively retrieved from the MOSAIQ database using Structured Query Language (SQL). A total of 275 patients with 4969 treatment fractions, treated within a span of 6 months, were included. The treatment sites of these patients included the brain, head and neck (HN), breast, lung, abdomen, pelvis, and extremity.

## 3 RESULTS

**Figure 4:**
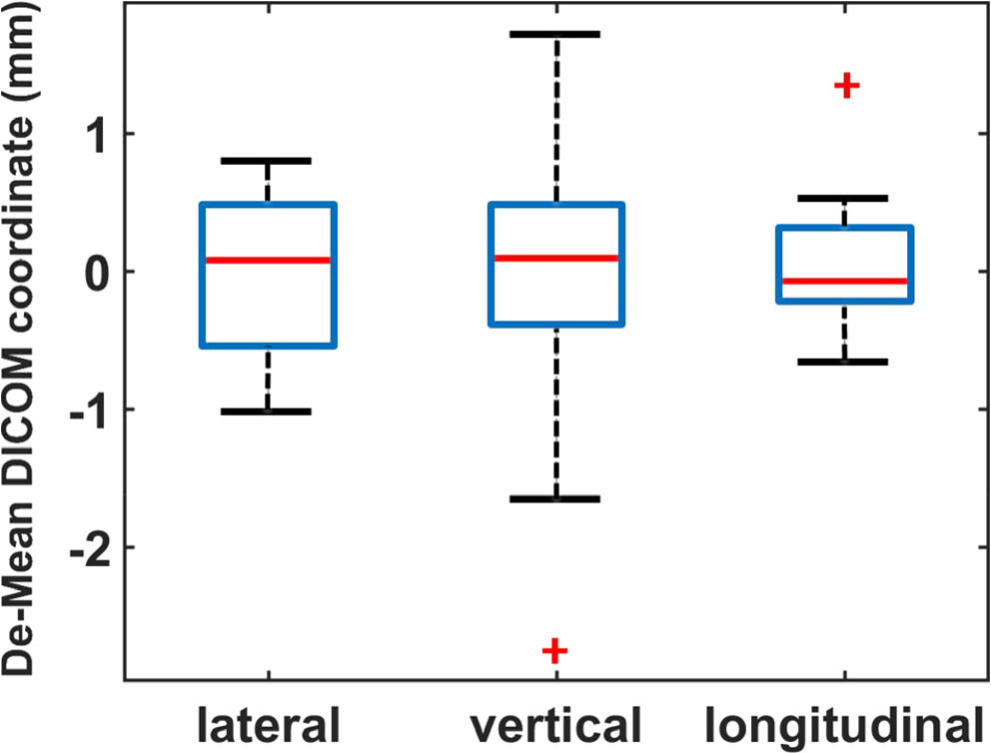
Uncertainty of the CT scanner’s DICOM coordinate system evaluated by scanning the CT couch‐top at various vertical and longitudinal positions. The central mark of the box plot indicates the median, and the bottom and top edges of the box indicate the 25th and 75th percentiles, respectively. The whiskers extend to the most extreme data points not considered outliers, and the outliers are plotted individually using the “+” symbol.

The uncertainty of the CT scanner’s DICOM coordinate system, evaluated without a patient on the treatment couch, is depicted in Fig. 4 using a box plot. The result is represented by the variation of BB3’s DICOM coordinates. The mean value, (0.0, −214.9, 974.3) mm, was subtracted out from the data. The variation was within 2.0 mm in the lateral and longitudinal directions and under 5.0 mm in the vertical direction.

Table 1 shows the uncertainty of the CT scanner’s DICOM coordinate system evaluated with the patient on the treatment couch. A total of 361 CT scans were reviewed. The number of CT scans using Head BB, BB1, BB2, BB3, and BB4 as the reference BB were 94, 22, 104, 131, and 10, respectively. BB5 (the most inferior reference BB) is excluded from the remaining discussion since it was not selected as a reference BB in any of these CT scans. The mean coordinates of all of the reference BBs in the lateral and vertical directions were within 1.0 mm and 5.0 mm, respectively; the longitudinal distance between adjacent reference BBs, evaluated by the difference between the mean longitudinal coordinates, agreed with the physical distance to within 1.0 mm. These results validated our observation that the DICOM coordinate system is defined by the couch‐top. In the lateral direction, BB4 had the smallest uncertainty range of 1.5 mm, and the other reference BBs had a similar uncertainty range of approximately 4.0 mm. The vertical direction showed the most significant variation: the head BB had an uncertainty range of 9.7 mm, while the other reference BBs had uncertainty ranges between 5 mm and 9 mm. In the longitudinal direction, the uncertainty range of all of the reference BBs was between 3.5 mm and 6.0 mm. These results have two implications: (1) the uncertainty of the DICOM coordinate system‐based couch coordinate calculation method can be up to nearly 10.0 mm in the vertical direction, depending on which reference is used to establish the mapping relationship; based on this result, we use a tolerance of 10.0 mm when comparing the calculation results of the two calculation methods and (2) to use the reference BB’s longitudinal DICOM coordinate to automatically identify which BB the reference BB is, a tolerance of 10.0 mm is sufficient. For example, if its longitudinal coordinate is between 964.3 mm and 984.3 mm, the reference BB can be correctly identified as BB3.

**Table 1:** Uncertainty of the CTs canner’s DICOM coordinate system evaluated with patient CT scans. In each scan, only the reference BB selected for the couch coordinate calculation is included in the analysis. For comparison, its uncertainty represented by BB3 without a patient on the couch is also included (last row).

Reference BB set	Lateral direction		Vertical direction		Longitudinal direction	
	Mean ± SD (mm)	(max‐min) (mm)	Mean ± SD (mm)	(max‐min) (mm)	Mean ± SD (mm)	(max‐min) (mm)
Head BB (N = 94)	0.7 ± 1.0	3.6	−221.7 ± 1.7	9.7	274.2 ± 1.0	4.5
BB1 (N = 22)	0.3 ± 1.1	4.0	−218.6 ± 1.6	6.5	693.2 ± 1.2	3.5
BB2 (N = 104)	0.5 ± 0.9	4.0	−218.0 ± 1.7	8.8	834.2 ± 1.1	5.3
BB3 (N = 131)	−0.3 ± 0.9	4.1	−217.9 ± 1.8	7.8	973.3 ± 1.2	6.0
BB4 (N = 10)	−0.2 ± 0.5	1.5	−216.1 ± 1.6	5.5	1184.1 ± 1.1	3.5
BB5 (N = 0)	–	–	–	–	–	–
BB3 (No patient)	0.0 ± 0.6	1.8	‐214.9 ± 0.9	4.5	974.3 ± 0.4	2.0

Abbreviations: N = Number of patients; SD = standard deviation.

**Figure 5:**
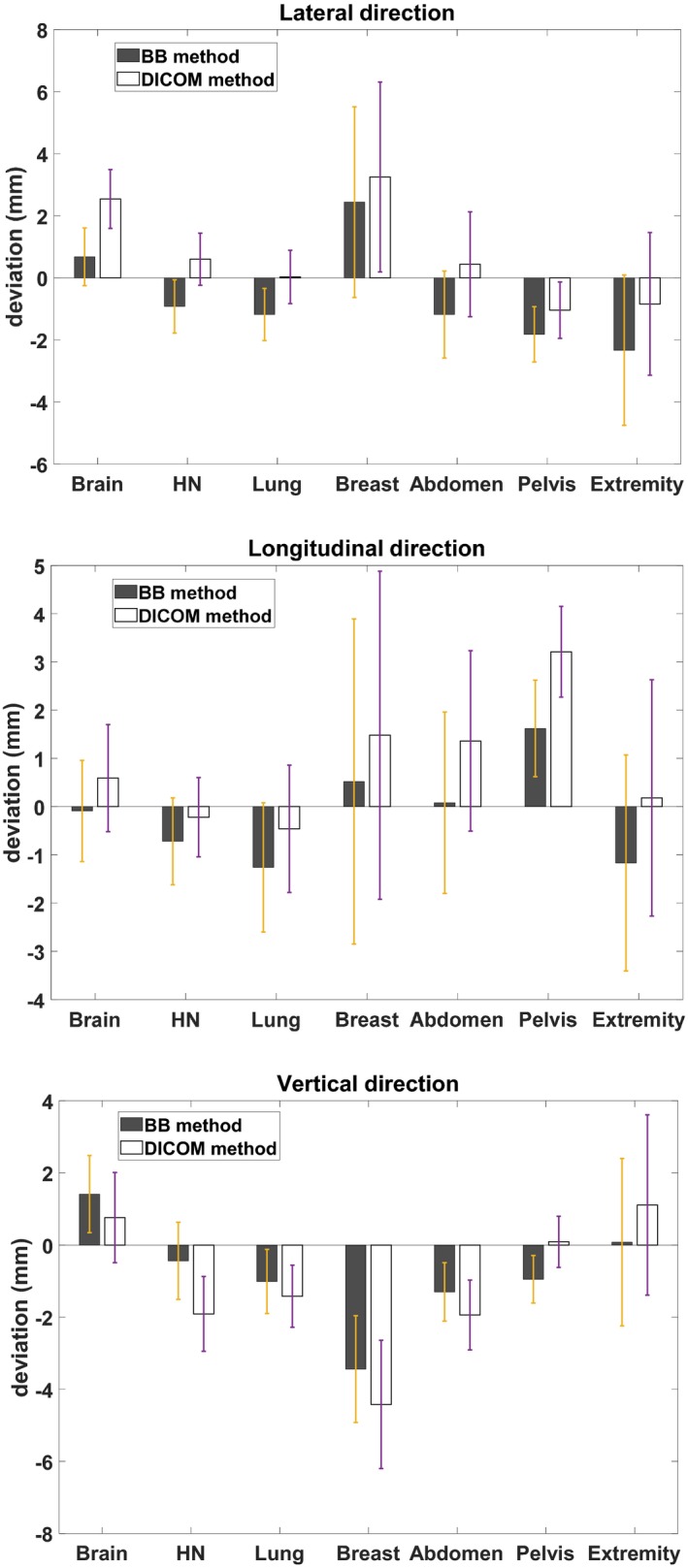
Accuracy of the two calculation methods, evaluated with treatment couch coordinates determined with cone‐beam tomography (CBCT) on the first day of treatment. The whisker indicates the 95% confidence interval.

Figure 5 shows the accuracy of both methods when compared to the final TCCs determined using CBCT on the first treatment day. The bars and error bars represent the mean deviation and the 95% confidence interval (CI), respectively. The analysis included 22 brain, 39 head and neck, 72 lung, 25 breast, 40 abdomen, 65 pelvis, and 12 extremity cases. The calculation was accurate, with 95% of the coordinate calculations within 3mm. Overall, with both methods, the most substantial average deviations were observed for the breast cases in the lateral and vertical directions and for pelvis cases in the longitudinal direction. This can be attributed to the fact that the treatment targets for these cases are inside soft tissue. This is especially obvious for patients with large or pendulous breasts. For the same reason, breast, abdomen and pelvis cases had the most significant error bars. Extremity cases also had slightly larger error bars, probably due to the increased difficulty in immobilization and the decreased setup reproducibility. The brain and head and neck cases had the best accuracy with variations to within 5 mm in all directions. It is relatively easy to reproduce the setup for these disease sites. Surprisingly, lung cases had good accuracy which was similar to that of the brain and head and neck cases. The overall accuracy of the reference BB‐based and DICOM coordinate‐based methods was comparable. The most substantial difference between the two methods was observed with the pelvis cases in the longitudinal direction, where the average deviation of the DICOM coordinate‐based method was 1.6 mm higher than that of the reference BB‐based method.

**Figure 6:**
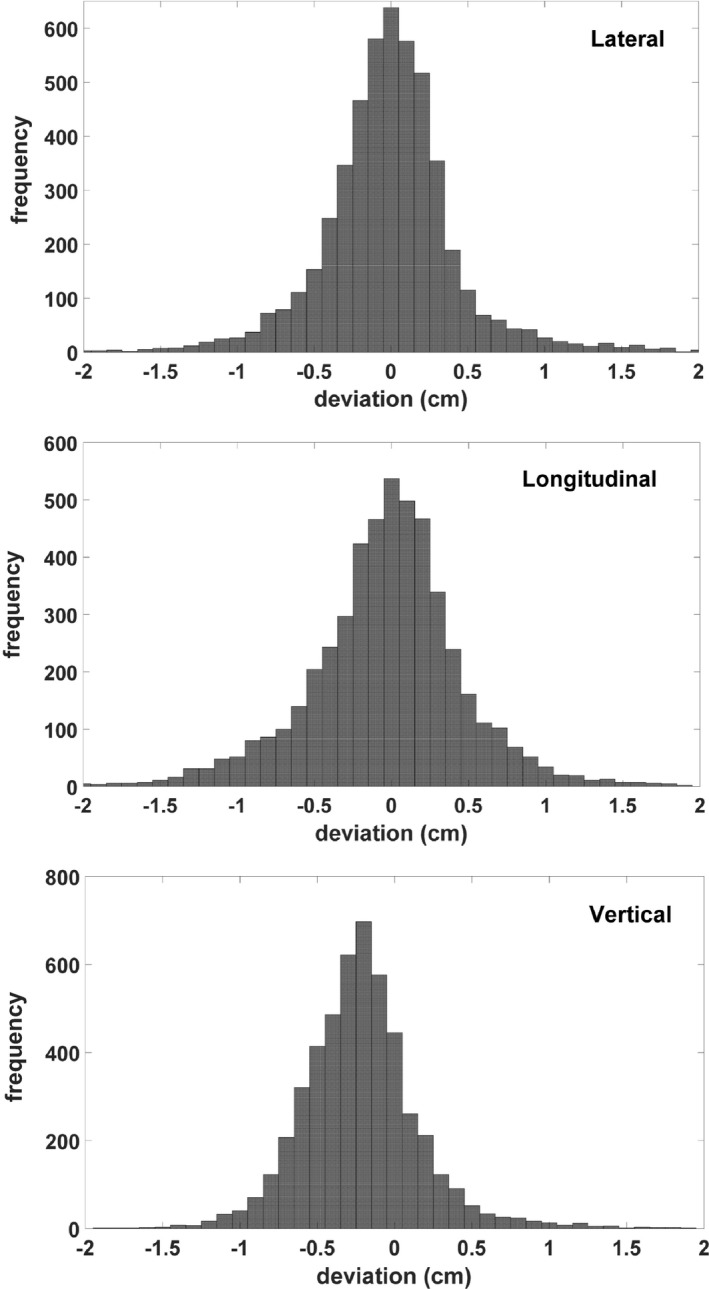
Deviation of the calculated treatment couch coordinates (TCCs) over the full treatment course. At each fraction, the patients were initially set up with the calculated TCCs then localized with daily CBCT. Reported here are the daily shifts from CBCT registration.

Figure 6 shows the histograms of inter‐fraction deviations for all of the cases combined over the whole treatment courses. Note the patients were initially set up using TCCs calculated with the reference BB‐based method, then localized with daily CBCT. The deviations reported here were essentially the shifts reported from CBCT registration. Ninety‐five percent and 99% of the coordinates were within ±1.05 cm and ±1.5 cm in the lateral direction, ±1.03 cm and ±1.56 cm in the longitudinal direction, and ±0.49 cm and ±0.87 cm in the vertical direction, respectively. A trend of increasing deviation was observed, which is probably due to the anatomical changes related to weight loss or tissue deformation, or organ motion related to respiration, rectal/bladder filling, etc. These results highlight the importance of daily CBCT in high‐precision radiotherapy.

## 4 DISCUSSION

In this work, we proposed two TCC calculation methods: the reference BB‐ and the DICOM coordinate‐based methods. These methods have clear advantages and disadvantages in terms of clinical implementation and workflow. The reference BB‐based system uses BBs that are permanently embedded in the couch‐top as the reference. Using these BBs has several advantages over using landmarks on immobilization devices as described by Saenz et al.^5^ First, not all immobilization devices have radiopaque landmarks that can be easily recognized on a CT scan. Second, the landmarks on different immobilization devices are most likely different in both location and appearance on the CT scans, which make it difficult for TPS users to correctly identify them. Third, the reference TCCs vary from one type of immobilization device to another, adding complexity to the overall workflow. Being independent of the immobilization device, the BBs physically embedded in the couch‐top overcome all of these difficulties. The unique arrangement of the embedded BBs makes it easy to distinguish between them, reducing the odds of misidentification. BB5 has never been used as a reference for any of our clinical CT scans since any extremity case that may have required their use was scanned feet‐first into the CT scanner bore. The downside of this approach is the need to modify the couch‐top by embedding multiple BBs. The DICOM coordinate‐based method is more straightforward since it does not use references for individual patients. The mapping relationship from the DICOM coordinate system to the TCC system can be established with a BB temporarily taped on the CT couch‐top. Therefore, unlike the reference BB‐based method, the DICOM coordinate‐based method does not need couch‐top modification. An additional advantage of the DICOM coordinate‐based method is the further simplification of the workflow. While the reference BB‐based method requires the TPS user to select a reference BB for each patient, the DICOM coordinate‐based method is transparent to the TPS user. This workflow simplification helps to avoid the potential user‐introduced error associated with the reference BB‐based method, where the reference BB could be incorrectly specified in the in‐house software. The potential problem with the DICOM coordinate‐based method is that it relies on its definition remaining stationary to the CT couch‐top, which can be changed unknowingly when the reset button is accidentally pressed during normal operation. Such incidents are unacceptable as the resultant errors will not be detected until another method (e.g., image‐based patient localization) is used to verify the patient’s position. In contrast, the reference BB‐based method does not have this problem. In summary, the two methods have equally excellent accuracy when used separately; however, they leave room for potential errors. Stringent QA measures (e.g., physics double‐check) need to be in place when a single method is used.

The combination of the two methods gives a robust self‐checking system along with a streamlined workflow. The CT DICOM coordinate system is used to facilitate the automatic identification of the reference BB. The self‐checking is performed by comparing the calculations from both methods. This design can detect nearly all of the error sources analyzed above. The accidental reset of the CT DICOM coordinate system leads to an offset in the DICOM coordinates. The software will similarly detect such an offset. The amount of offset can be determined from the difference between a reference BB's known DICOM coordinates and its DICOM coordinates as observed on the CT scan. This offset is then fed into the software to complete the reference BB identification and calculation of the couch coordinates. The CT DICOM coordinate system can then be reset to its correction position.

The design of the combined method emphasizes the workflow transparency and simplification via automation. Traditionally, the therapists make tattoos on the patient’s skin during CT simulation; the dosimetrists mark the BBs representing the location of the tattoos in the TPS and transfer the shift instructions to the therapists for treatment; for the treatment, the therapists perform a simple calculation to determine the couch coordinates while the patient is on the treatment couch. This process requires human involvement at each stage, leaving a few pathways for potential errors. In contrast, our workflow is nearly transparent to the therapists at CT simulation and treatment. They can focus their attention on achieving a proper immobilization and patient care. As for the dosimetrists, their sole responsibility is to find a reference BB near the treatment area where the laser coordinate system origin is placed. Workflow simplification via automation plays a vital role. For example, the process to create the point of interest for the reference BB and to transfer the DICOM coordinates from the TPS to the in‐house software can be simplified as single button clicks by an embedded software “script,” created with the Pinnacle system scripting language. This eliminates the need to type the numbers manually, which is a typical source of error. Similarly, the automatic identification of the reference BB in the in‐house software via its DICOM coordinates simplifies the workflow and obviates a potential error pathway. Currently, the calculated couch coordinates need to be manually entered into the R&V system. The automation of this step involves writing data directly into the R&V database, which is not supported by the vendor. We envision that if the TPS vendor adopts the proposed method, the couch coordinates can be calculated by the TPS and directly transferred to the R&V system along with other beam parameters (e.g., gantry angle, number of monitor units, etc.). At present, these manually entered coordinates are rigorously checked against the report generated by the in‐house software by the physicist during the initial chart check. It is worth mentioning that the calculated TCCs are only used for initial patient setup. The final treatment position is determined with subsequent daily CBCT.

As part of our continuous quality improvement efforts, two QA measures have been implemented to prevent or detect an accidental reset of the CT DICOM coordinate system. First, a ring‐shaped device that was custom‐built with 3D printing has been mounted on top of the CT scanner reset button. If the CT scanner needs a reset, a pen‐like object must be used to reach the reset button to press it. Second, as part of the daily CT scanner QA, the CT couch‐top is moved to align a pre‐marked position with the wall laser. The reading of the couch‐top’s longitudinal position is checked against the reference value using a 1.5 mm tolerance. After the clinical implementation of the DICOM method a year ago, but prior to the introduction of these QA measures, the CT DICOM coordinate system was accidentally reset twice within 2 months. An accidental reset has not occurred since the QA measures were put into place. The assumption that the DICOM coordinate system is defined with respect to the CT couch‐top plays a vital role in the proposed method. Although we validated this assumption on our Phillip Brilliance Big Bore CT simulator, we are not sure whether this feature is common across CT scanners from different vendors since we have no access to other CT scanners. However, it is straightforward to check on any CT scanners using our validation method, that is, tape a BB on the couch‐top, perform repeated CT scans with different initial couch height and longitudinal positions, and check whether the TPS‐reported DICOM coordinates of the BB remain the same.

Note that the proposed system does not rely on the use of skin tattoos or room lasers for patient localization. However, they are used for patient’s rotational correction, especially for patients treated with body mold. Two sets of tattoos are marked on the patient skin at two different axial planes near the abdominopelvic area (one on top and one on either side), as conventionally done under the guidance of the CT scanner’s laser system. The longitudinal locations of the tattoos are marked on the body mold. During the treatment, patient rotational errors can be corrected by aligning the two sets of tattoos with treatment room laser in sequence. The therapists also align the skin tattoos with the marks on the body mold to ensure that the patients reproduce their positions.

For inter‐fractional deviations over the entire treatment course in Fig. 6, the bell‐shaped histograms were centered around zero in the lateral and longitudinal directions, but a 0.25 cm shift toward the negative direction was found in the vertical direction. This was consistent with the results from day 1 as shown in Fig. 5. The deviations for most cases (except brain and extremity) in the vertical direction were negative, while they were more balanced in the other two directions. This was probably due to the increased couch sag with the linear accelerator as compared with the CT scanner. We have observed increased, albeit small couch sag as the treatment couch moves toward the gantry while the patient is on the couch. The clinical impact of this vertical shift toward the negative direction was negligible since the final treatment position was determined with CBCT where the registration was completed based on the actual treatment target.

In addition to preventing near‐miss incidents for patient treatments, there are three additional benefits of being able to determine the couch coordinates in advance. The first one is related to a junction area on the treatment table that has a significantly higher beam attenuation than the rest of the table. Based on the calculated couch coordinates, we can tell whether the treatment beam will pass through the junction area. If needed, different indexing holes can be used to shift the immobilization device relative to the table in order to move the junction out of the beam path. By our approach, this decision can be made in the treatment planning stage, instead at the time of treatment. The resultant offset is accounted for by adjusting the longitudinal couch coordinate (TCC_z_) correspondingly. The second benefit is the avoidance of possible couch collisions. For example, to treat targets located significantly off the patient’s midline, large lateral couch offset is needed, which can lead to collision between the couch and the gantry. This can be determined in advance using the calculated couch coordinates, and the treatment planning isocenter can be shifted toward the patient’s midline to avoid the collision. The third benefit is that the software can determine whether the TCCs exceed the couch’s limit. If it occurs, the system offers the opportunity to remedy the situation by nondefault indexing holes or adjusting treatment planning isocenter position. These are all benefits that allow us to foresee and prevent the potential events that, using conventional setup methods, would only be discovered after the patient is on the treatment couch.

## 5 CONCLUSION

We have proposed two TCC calculation methods and a highly automated self‐checking system. Combining the use of reference BBs physically embedded in the couch‐top and the CT scanner’s DICOM coordinates, the system calculates TCCs with excellent accuracy in advance. The system universally applies to all treatment sites with any indexed immobilization devices. A simple, robust, streamlined workflow is achieved via automation, eliminating nearly all of the common error pathways. The implementation of the system significantly eases the pressure on the therapists at the time of treatment, reduces patient setup time, and enhances the patient safety by minimizing the chance of medical error events related to patient setup.

## CONFLICT OF INTEREST

The authors have no conflict of interest to declare.
